# Lipid-lowering activity of Cow urine ark in guinea pigs fed with a high cholesterol diet

**Published:** 2014

**Authors:** Chawda Hiren Manubhai, Mandavia Divyesh Rasiklal, Baxi Seema Natvarlal, Vadgama Vishalkumar Kishorbhai, Tripathi ‎Chandrabhanu Rajkishor

**Affiliations:** 1 *Department of Pharmacology, Government Medical College, Bhavnagar-364001, Gujarat, India*; 2 *Department of Pathology, Government Medical College, Bhavnagar-364001, Gujarat, India*

**Keywords:** *Antioxidantactivity*, *Cowurineark*, *Dyslipidemia*, *Hypolipidemia*, *Guineapig*, *Statin*

## Abstract

**Objectives:** Cow urine ark (CUA), known as “Amrita” as mentioned in Ayurveda, contains‎ anti-hyperglycemic and antioxidant effects. Therefore, we designed the present study to evaluate the lipid ‎lowering activity of CUA and its possible implication in metabolic syndrome.‎

**Materials and Methods:** Thirty guinea pigs of either sex were divided into five groups: Group 1 and 2 serving as a vehicle ‎and sham control, received normal and high fat diet for 60 days respectively; Group 3, 4 and 5 ‎received high fat diet for 60 days with CUA 0.8 ml/kg, 1.6 ml/kg and rosuvastatin (1.5 mg/kg) on the‎last 30 days of study period, respectively. Serum lipid profile (total cholesterol, triglycerides, LDL-‎C, VLDL-C, HDL-C, total Cholesterol/HDL-C) and serum enzymes (ALT, AST, ALP, LDH and CK-MB) ‎were performed in each group at the beginning and end of the study. Histological study of liver and ‎kidney was done in each group.

**Results: **CUA (0.8 ml/kg) significantly decreased the serum triglycerides and VLDL-C, but CUA (1.6 ml/kg) ‎decreased the total serum Cholesterol, triglycerides and VLDL-C (p < 0.05). Higher dose (1.6 ml/kg) of ‎CUA also increased HDL-C level, significantly (p < 0.05). CUA reduced serum AST, ALP and LDH ‎level, which was statistically significant as well, while it also decreased the accumulation of lipid in hepatocytes as ‎compared to sham control.‎

**Conclusions:** CUA reduced triglycerides, increased HDL-C and found to be hepatoprotective in ‎animals that are on a high fat diet.

## Introduction

Cardiovascular diseases (CVD) are emerging problem in developing countries ‎‎(Freedman, 2003 ▶; Badimon et al., 2010 ▶). CVD may become the reason for the loss of 17.9 ‎million Potentially Productive Years of Life Lost (PPYLL) in India, by 2030 (Anchala et al., 2012 ▶). ‎Dyslipidaemia is well recognized risk factor for the development of cardiovascular diseases ‎‎(Freedman, 2003 ▶; Badimon et al., 2010 ▶). Reactive oxygen species induce the oxidative stress, ‎which play significant role in the development of CVD and atherosclerosis. Liver, kidney ‎and heart are also under oxidative stress due to hyperlipidemia in CVD (Kwiterovich, ‎‎1997 ▶; Vijayakumar et al., 2004 ▶). Other crucial factors regarding CVD, associated morbidity and mortality, are the insulin resistance, diabetes, ‎elevated blood pressure, obesity and dyslipidaemia that are included in metabolic syndrome (Li et al., 2013 ▶). Modern life style like smoking, alcohol ‎consumption and junk food diet are major obstacles in the management of dyslipidaemia. ‎Elevated liver enzymes are the major risk factors associated with the current allopathic treatment ‎for dyslipidaemia and thus, the alternative medicines from Ayurveda, have been getting attention ‎in the management of dyslipidaemia (Suanarunsawat et al., 2011 ▶). ‎

Cow urine, known as “Amrita” or water of life as mentioned in Ayurveda ‎‎(Dhama et al., 2005 ▶), is one of the ingredients of Panchagavya Ghrita. Panchagavyapathy was proved to be effective in life threatening diseases such as diabetes, cancer and AIDS ‎‎(Dhama et al., 2005 ▶; Achliya et al., 2003 ▶; Jarald et al., 2008 ▶). Cow urine has medicinal ‎properties like antimicrobial, antifungal and anticancer which granted US Patents (No. ‎‎6,896,907 and 6,410,059) (Randhawa, 2010 ▶). Several studies revealed that cow urine has ‎antidiabetic and antioxidant activity (Sachdev et al., 2012 ▶; Krishnamurthi et al., 2004 ▶). ‎Therefore, the present study was planned to evaluate the lipid lowering effect of Cow urine ark ‎‎(CUA) and also to know its possible implication in metabolic syndrome.‎

## Materials and Methods

**Drugs and Chemicals**

Cholesterol powder (analytical grade): was purchased from High Purity Laboratory 

Chemicals ‎Pvt. Ltd., Mumbai, India. Cow urine ark (CUA) was obtained from Go Vigyan‎Anushandhan Kendra, Sevadhanm, Devalpur, Nagpur, Maharashtra, India and (US Patent ‎No 6410 059/2002). Rosuvastatin Calcium powder: (Gift sample from Torrent ‎Pharmaceuticals Ltd., Torrent research center, Ahmedabad. (Batch no: ARD2110109)‎

**Animal preparation**

Thirty guinea pigs weighing 520 – 860 g of either sex were procured from the Central ‎Animal House of Government Medical College, Bhavnagar, which is registered in the ‎Committee for the Purpose of Control and Supervision of the Experiments on Animals ‎‎(CPCSEA), New Delhi, India. CPCSEA guidelines were followed during animal ‎experiments of our study. The guinea pigs were housed in stainless steel cages under 12 ‎hour light-dark cycle room with controlled temperature at 23±2°C, being fed with standard ‎laboratory food and water ad libitum. After the proper acclimatization for 15 days, they were ‎divided into five groups of six guinea pigs.‎

Group I: Normal diet plus distilled water (Vehicle control); Group II: High fat diet plus ‎distilled water (Sham control); Group III: High fat diet plus a lower dose of CUA (0.8 ‎ml/kg); Group IV: High fat diet plus a higher dose of CUA (1.6 ml/kg); Group V: High fat ‎diet plus rosuvastatin (1.5 mg/kg)‎

**Diet composition** **‎**

Normal diet: in the morning, mixtures of cereals and pulses (60% wheat plus 35% Bengal ‎gram plus 15% peanuts), total 50 g/animal. In the evening: green leafy vegetables, 30 g/ ‎animal.‎ High fat diet: in the morning, cholesterol powder (500 mg/kg) mixed with 10 g of wheat and ‎Bengal gram flour, followed by 40 g of the mixtures mentioned above, in the normal diet/animal. ‎In the evening: green leafy vegetables, 30 g/animal.‎

**Methodology**

The study was conducted in Animal room, Department of Pharmacology, Government ‎Medical College, Bhavnagar, Gujarat, after approval from the Institutional Animal Ethics ‎Committee of the same institute. The baseline blood sample was collected from a lateral ‎saphenous vein of the hind paw of each guinea pig in overnight fasting state. Blood samples were‎ sent to Clinical Biochemistry Laboratory of our institute which is accredited by National ‎Accreditation Board for Testing and Calibration Laboratories (NABL), for the serum lipid ‎profile, liver and cardiac enzymes. Animals were separated into groups as mentioned above. Throughout the study period in each group, diet ‎was given according to the respective group diet plan. During the last 30 days of the experiment, animals of group I and II ‎were given distilled water daily, animals of group III, IV and IV were given a lower dose ‎of CUA, higher dose of CUA and rosuvastatin (1.5 mg/kg), respectively. ‎Distilled water, CUA and rosuvastatin calcium were given orally by gavages feeding tube ‎in a daily basis on mornings in fasting state to ensure maximum absorption. Animals of all groups ‎were sacrificed after blood collection from the lateral saphenous vein in the overnight fasting ‎stat at the end of 60 days. Bloodsmples were sent to labratory for the analysis of the serum lipid profile, liver and ‎cardiac enzymes. We obtained the liver and kidney from each animal of the five groups ‎for histopathological analysis, which was done by senior faculty from the Pathology ‎department of our institute.‎

**Serum lipid profile**

The serum levels of triglycerides, total cholesterol and high density lipoprotein cholesterol ‎‎(HDL-C) and Low density lipoprotein cholesterol (LDL-C) were analyzed by GPO PAP ‎METHOD, CHOD PAP METHOD, IMMUNOINHIBITION and ENZYME SELECTIVE ‎methods, respectively. Very low density lipoprotein cholesterol (VLDL-C) was calculated ‎by the Friedwald method (Friedewald et al., 1972 ▶) as well as the ratio of the total cholesterol and HDL-C.‎

**Evaluation of liver and cardiac enzymes**

Liver function was evaluated by serum alanine aminotransferase (ALT), aspartate ‎aminotransferase (AST), and alkaline phosphatase (AP) levels. Cardiac injury was assessed ‎by measuring the serum level of creatine kinase MB subunit (CK-MB) and lactate ‎dehydrogenase (LDH). ALT and AST were examined by UV KINETIC method and serum ‎alkaline phosphatase was determinedby PNP AMP KINETIC method. LDH and CK-MB ‎were analyzed by IMMUNO-INHIBITION and UV KINETIC, respectively.‎

**Effect of CUA on the weight of the animals**

Weighing of each animal in all groups was done before the start of the study and also at the ‎end of 60 days to rule out any effect of CUA on the weight of the guinea pig.‎

**Histological analysis**

The liver and kidney were isolated, cleaned, dried, and fixed at 10% neutral buffer formalin ‎followed by paraffin embedding and stained with haematoxylin and eosin (H&E) dye. All ‎histopathological slides were coded and evaluated by a pathologist blindly without ‎knowledge of the groups. Scalinggrades 0, 1, 2, 3 and 4 were given for no change, slight, mild, ‎moderate and severe changes, respectively regarding theseverity assessment of histopathological‎ results.‎

**Statistical analysis:** **‎**

All parameters were expressed as Mean±standard error of mean (S.E.M.). One-way ‎Analysis of Variance (ANOVA) followed by Tukey-Kramer Multiple comparison test was ‎used to compare the inter group differences of lipid profile, liver enzymes, cardiac enzymes and ‎extent of body weight gain at the end of 60 days. Paired t-test was used to compare intra ‎group differences of lipid profile, liver enzymes, cardiac enzymes and extent of body weight ‎gain. Value of p<0.05 was considered significant. The statistical calculations were done ‎using GraphPadInStat, Demo version 3.06.‎

## Results

**Serum lipid profile**

The baseline serum level of total cholesterol, triglyceride, HDL-C, LDL-C ‎and VLDL-C are 46.33 ± 4.04, 92 ± 5.24, 4.5 ± 0.9, 23.56 ± 2.6 and 19.2 ± 1.16, ‎respectively in vehicle control (Table 1). There are not statistically significant differences in the ‎baseline values of each variable in different treatment groups. The serum level of the total ‎cholesterol, triglyceride, HDL-C, LDL-C and VLDL-C were 82.6 ± 4.65, 109.2 ± 7.63, 3.9 ‎‎± 0.8, 54.38 ± 7.1 and 22.5 ± 3.5, respectively at the end of 60 days, which were statistically ‎significant as compared to the baseline lipid profile, in Sham control [p<0.05; Table 1]. The percentages of increment in serum level of the total cholesterol, triglyceride, HDL-C, ‎LDL-C and VLDL-C were 92 ± 22.8 (%), 29.8 ± 7.5 (%), 63.2 ± 45.4 (%), 161.93 ± 45.5 (%) ‎and 29.8 ± 7.5 (%) in sham control, as showed in Table ‎‎2.‎

A significant reduction in serum triglyceride and VLDL-C was observed in high fat diet treated group with the lower dose of cow urine ark (CUA), in comparison to sham control. The serum total cholesterol, serum triglyceride and VLDL-C were ‎significantly decreased in the higher dose of CUA as compared to sham control [p<0.05; ‎‎(Table 1)]. However, the HDL-C level was significantly increased with higher dose of CUA (p < 0.05) but, ‎percentage of increment in HDL-C with rosuvastatin treatment was significantly greater than ‎the lower and higher dose of CUA treatment. The percentage of increment in ‎triglyceride and VLDL-C with rosuvastatin treatment were- 8.82 ± 2.3 and - 8.8 ± 2.3, ‎respectively at 60 days, while with lower ‎dose of CUA and higher dose of CUA, the percentage results were - 26.5 ± 12.7 and - 37.1 ± 8.2, (Table 2).‎

**Table 1:** Effect of each treatment strategy on serum lipid profile in guinea pigs

**Treatment Groups** **(n = 6)**	**Time Period**	**Total Cholesterol** **(mg/dl)**	**Triglycerides** **(mg/dl)**	**HDL Cholesterol** ** (mg/dl)**	**LDL Cholesterol** ** (mg/dl)**	**VLDL Cholesterol** ** (mg/dl)**	**Total Cholesterol/** **HDL-C **
**Vehicle control** **(Group 1)**	Base line	46.33 ± 4.04	92 ± 5.24	4.5 ± 0.9	23.56 ± 2.6	19.2 ± 1.16	11.4 ± 0.8
	60 Days	46.53 ± 5.02	88.7 ± 6.65	4.4 ± 0.5	24.17 ± 2.13	18.7 ± 1.22	12.02 ± 0.6
**Sham control** **(Group 2)**	Base line	46.2 ± 7.03	87.38 ± 6.2	4.1 ± 0.8	23.26 ± 3.47	17.3 ± 1.24	9.78 ± 1.5
	60 Days	82.6 ± 4.65**	109.2 ± 7.63**	3.9 ± 0.8	54.38 ± 7.1**	22.5 ± 3.5**	15.39 ± 7.48
**High fat diet plus CUA (0.8 ml/kg)** **(Group 3)**	Base line	47.8 ± 3.43	75 ± 9.25	4.5 ± 0.6	28.33 ± 2.7	15 ± 1.85	12.53 ± 3.19
	60 Days	64.9 ± 7.92	49.5 ± 2.65*	7.16 ± 1.6	47.6 ± 6.4	9.9 ± 0.5*	10.44 ± 1.86
**High fat diet plus CUA (1.6 ml/kg)** **(Group 4)**	Base line	46.23 ± 2.24	75.3 ± 6.01	4.3 ± 0.42	26.83 ± 4.26	15.06 ± 1.2	11.2 ± 1.45
	60 Days	57.53 ± 2.9*	45.3 ± 3.21*	6.3 ± 0.6**	42.1 ± 1.9	9.06 ± 0.64*	9.29 ± 0.52
**High fat diet plus rosuvastatin** **(1.5 mg/kg)** **( (Group 5)**	Base line	42.09 ± 8.64	86.83 ± 2.14	5.8 ± 0.7	26 ± 2.57	15.57 ± 2.3	8.8 ± 0.6
	60 Days	46.32 ± 5.21*	76.42 ± 4.6*	9.6 ± 0.9*	28 ± 5.15*	15.08 ± 2.3*	4.55 ± 0.65*

Values are expressed as Mean ± standard error of mean; LDL: low density lipoprotein, VLDL: very low density lipoprotein, HDL: high density lipoprotein; CUA: Cow urine ark;

* p< 0.05 as compared to sham control, ANOVA followed by Tukey-Kramer Multiple comparison test;

** p< 0.05 as compared to baseline level, paired *t*-test.

**Table 2:** The effects of each treatment strategy on serum lipid profile (% increment) on guinea pigsat the end of 60 days treatment

**Treatment Groups(n = 6)**	**Time Period**	**Total Cholesterol** **(mg/dl)**	**Triglycerides** **(mg/dl)**	**HDL Cholesterol** **(mg/dl)**	**LDL Cholesterol** **(mg/dl)**	**VLDL Cholesterol** **(mg/dl)**	**Total Cholesterol/** **HDL-C **
**Vehicle control ** **(Group 1)**	**60 Days**	- 2.4 ± 1.8	- 2.4 ± 1.8	- 2.4 ± 2.4	- 2.8 ± 3.8	- 2.3 ± 1.8	0.3 ± 3.4
**Sham control** ** (Group 2)**	**60 Days**	92 ± 22.8*	29.8 ± 7.5*	63.2 ± 45.4	161.93 ± 45.5*	29.8 ± 7.5*	54.2 ± 30.7
**High fat diet plus CUA (0.8 ml/kg) (Group 3)**	**60 Days**	42.9 ± 25.3	- 26. 5 ± 12.7#	70.8 ± 36##	78.9 ± 34.9	- 26.5 ± 12.7#	5.9 ± 30
**High fat diet plus CUA (1.6 ml/kg) (Group 4)**	**60 Days**	27.2 ± 8.5	- 37.1 ± 8.2#	50.5 ± 16##	72 ± 21.7	- 37.1 ± 8.2#	- 8.85 ± 13.2
**High fat diet plus Rosuvastatin** **(1.5 mg/kg) (Group 5)**	**60 Days**	4.5 ± 4.9**	- 8.82 ± 2.3**	234 ± 31**	- 18.9 ± 3.9**	- 8.8 ± 2.3**	- 67.9 ± 2.1**

Values are expressed as Mean ± standard error of mean; LDL: low density lipoprotein, VLDL: very low density lipoprotein, HDL: high density lipoprotein; CUA: Cow urine ark;

* p< 0.05 as compared to vehicle control, ANOVA followed by Tukey-Kramer Multiple comparison test;

** p< 0.05 as compared to sham control, ANOVA followed by Tukey-Kramer Multiple comparison test;

# p< 0.001 as compared to sham control, ANOVA followed by Tukey-Kramer Multiple comparison test;

## p< 0.05 as compared to rosuvastatin treatment group, ANOVA followed by Tukey-Kramer Multiple comparison test.

The baseline and 60 days values of serum enzymes in each diet treatment group were shown (Table3).In sham control, there is a significant increase in ALT, AST, ALP, LDH and CK-MB level (p<0.05) at the end of 60 days, compared to the baseline.ALT, AST and LDH levels were significantly decreased in the high fat diet group treated with low dose of CUA. However, LDH level was significantly decreased with the higher dose of CUA compared to sham control [p<0.05;(Table 3)].High fat diet treated group with rosuvastatin (1.5 mg/kg) showed significant reduction in the serum total cholesterol, LDL-C and ratio of total cholesterol: HDL-C level and an increase in HDL-C level as compared to sham control [p<0.05; (Table 1)]. Compared to the baseline level, there was an increase in AST and ALP level in the rosuvastatin treated group at the end of 60 days period[p<0.05; (Table 3)]. 

**Figure 1:**
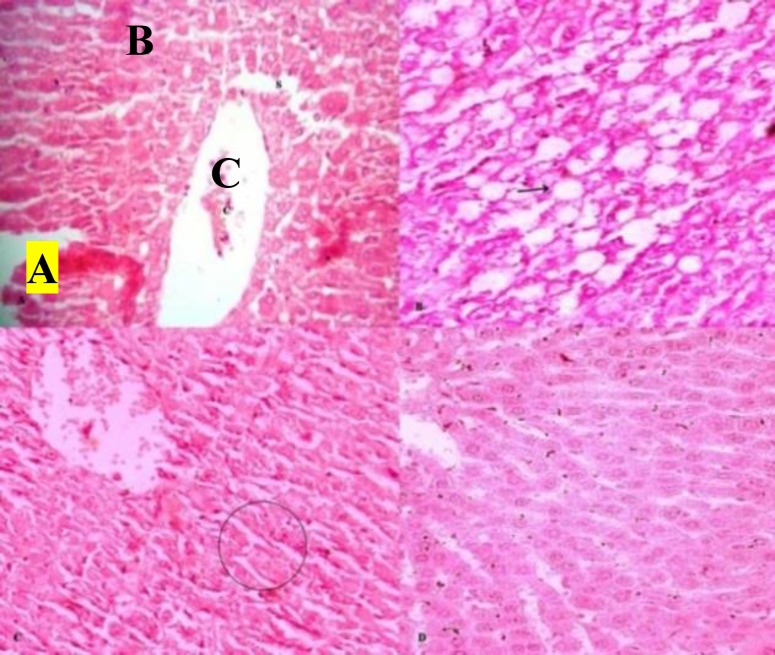
Histological photograph of liver of guinea pig (H&E 40x). (A) C, H and S indicates

**Figure 2:**
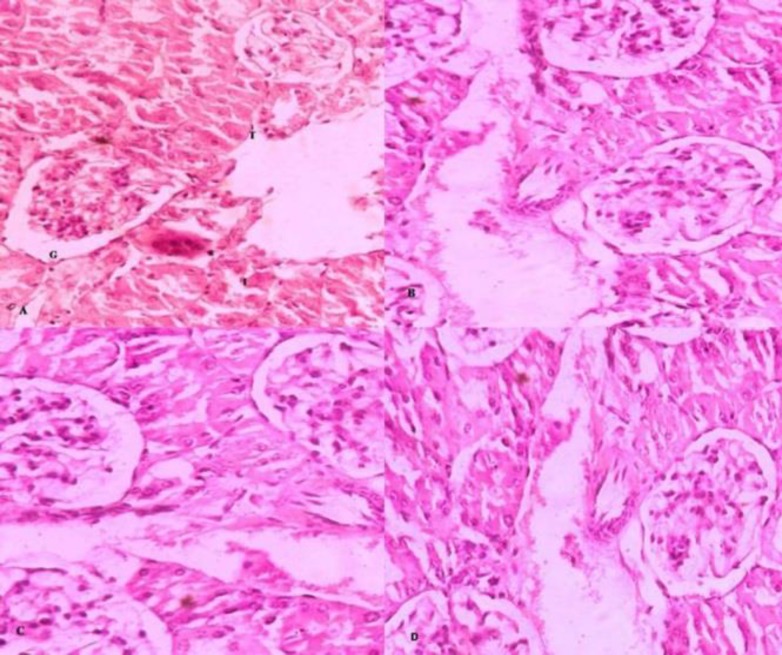
Histological photograph of kidney of guinea pig (H&E 40x). (A) G, T and I indicate normal structure of glomeruli, tubule and interstitum, respectively; (B) No fatty changes were seen in high fat diet fed guinea pig; (C) and (D) indicate normal histological appearance of kidney in CUA (1.6 ml/kg) and rosuvastatin (1.5 mg/kg) treated groups, respectively

The normal histological structure of liver and kidney was shown in Figure 1 A and 2 A, respectively. In sham control, histological study of the liver showed diffuse areas ballooning degeneration of hepatocytes (grade 3+, 4+), Steatosis (up to grade 2.5), fatty changes (midzone and periportal) and congestion of central vein and hepatic sinusoids (Figure 1 B) in all animals, while the kidneys showed a normal structure (Figure 2 B). In the group treated with CUA and high fat diet, liver histology showed no fatty changes, with only ballooning degeneration (Grade 1+) with repaired and regeneration process (Figure 1 C) and the kidney examination of this group were normal (Figure 2 C). In rosuvastatin treated group, no morphological changes in the liver and kidney was detected (Figure 1 D and Figure: 2 D).

Increase in mean body weight of all groups at the end of 60 days was shown ‎in Table 4 and the extent of weight gain was not statistically significant between the groups ‎‎(p<0.05).

**Table 3:** Effect of each treatment strategy on serum enzymes in guinea pigs

**Treatment Groups** **(n = 6)**	**Time Period**	**ALT** **(U/L)**	**AST** **(U/L)**	**ALP** **(U/L)**	**LDH** **(U/L)**	**CKMB** **(U/L)**
**Vehicle control** **(Group 1)**	**Base line**	53.46 ± 1.86	61.33 ± 7.95	94.8 ± 12.67	372.83 ± 44.03	268.8 ± 11.5
	**60 Days**	55.81 ± 2.86	63.33 ± 7.6	97.5 ± 9.5	377.16 ± 46.12	267.7 ± 17.6
**Sham control** **(Group 2)**	**Base line**	61.17 ± 6.21	50.33 ± 5.3	85.83 ± 11.7	311.5 ± 39.05	285 ± 34.74
	**60 Days**	104 ± 12.72**	162.4 ± 25.4**	145.6 ± 9.6^**^	456.5 ± 56.8^**^	360.23 ± 27.1^**^
**High fat diet plus CUA (0.8 ml/kg)** **(Group 3)**	**Base line**	57.14 ± 4.3	63.76 ± 14.9	126.5 ± 18.8	345.3 ± 22.6	433.56 ± 22.1
	**60 Days**	50.83 ± 9.9*	72.66 ± 22.1*	139.9 ± 9.07	194.16 ± 28.12^*^	353.8 ± 36.5
**High fat diet plus CUA (1.6 ml/kg)** **(Group 4)**	**Base line**	51 ± 1.3	73 ± 14.7	86.6 ± 15	283.6 ± 27.2	317.33 ± 22.5
	**60 Days**	63.8 ± 6.5	96.8 ± 14.6	124.3 ± 12.73	243.5 ± 37.4^*^	297.6 ± 17.5
**High fat diet plus rosuvastatin** **(1.5 mg/kg)** **(Group 5)**	**Base line**	63.26 ± 13.2	62.26 ± 9.65	103.6 ± 12.3	241 ± 46.64	374.5 ± 42.25
	**60 Days**	64.34 ± 5.5	169 ± 12.68**	161.5 ± 10.91^**^	301.8 ± 31.12	392.19 ± 28.3

Values are expressed as Mean ± standard error of mean; ALT: Alanine aminotransferase, AST: Aspartate aminotransferase,AP: Akaline phosphatase , LDH: Lactate dehyrogenase, CK-MB: Creatine kinase-MB, CUA: Cow urine ark;

* p<0.05 as compared to sham control, ANOVA followed by Tukey-Kramer Multiple comparison test;

** p< 0.05 as compared to baseline level, paired *t*-test.

normal structure of central vein, hepatocytes with round nucleus and sinusoid, respectively; (B) Fatty changes (Grade 4+) (arrow) and diffuse areas of ballooning degeneration were shown in high fat diet fed guinea pig; (C) Encircled area shown no fatty changes, only ballooning degeneration (Grade 1+) with repaired and regeneration process in CUA (1.6 ml/kg) treated group; (D) Normal morphological changes seen in rosuvastatin (1.5 mg/kg) treated group.

‎

**Table 4:** Effect of each treatment strategy on weight of guinea pigs

**Treatment group**	**Weight of animal in grams**	
	**Baseline**	**60 days**
**Vehicle control**	540.66 ± 6.14	554.16 ± 15.97
**Sham control**	632.33 ± 44.3	667.82 ± 45.32*
**High fat diet plus CUA (0.8 ml/kg)**	602.3 ± 29.3	637.6 ± 17.8*
**High fat diet plus CUA (1.6 ml/kg)**	604.3 ± 9.2	630.1 ±4.6*
**High fat diet plus rosuvastatin(1.5mg/kg)**	658 ± 36.8	682.5 ± 22.02*

**Values are** **expressed as Mean ± standard error of mean;** **CUA: Cow urine ark; **

* **p< 0.05 as compared to baseline value, paired** ***t*** **-test.**

## Discussion

In the present study, we selected guinea pig as the experimental animal for the ‎evaluation of lipid lowering activity of Cow urine ark (CUA). Lipoprotein metabolism of ‎guinea pigs is closest to human and several lines of evidence proved that guinea pigs are ‎admirable models to assess hypolipidemic activity and lipoprotein metabolism of drugs ‎‎(Fernandez and Volek, 2006 ▶).‎

The present study showed that 60 days of high cholesterol diet feeding, ‎raised the serum lipid profile (total cholesterol, triglyceride, LDL-C and VLDL-C) and ‎induced the histopathological changes in liver (Table 1, Figure 1 B). The liver plays a major role in ‎equilibrium cholesterol homeostasis (Suanarunsawat et al., 2011 ▶). High cholesterol diet ‎increases the hepatic cholesterol content and is responsible for the elevated triglyceride synthesis and ‎cholesteryl ester-rich VLDL-C production (Patel Y et al., 2011 ▶; Goldstein et al., 1983 ▶; ‎Demacker et al., 1991 ▶). The reduction in the amount ofhepatic LDL-‎C receptors is caused by high fat diet which diminishes cholesterol removal rate from it (Patel et al., 2011 ▶; Goldstein et al., ‎‎1983 ▶; Demacker et al., 1991 ▶). We opted a study period, 60 days, which is sufficient to ‎produce fatty changes in guinea pigs, also supported by previous studies (Patel et al., 2011 ▶; ‎Ahmad-Raus et al., 2001 ▶). ‎

In the present study, CUA therapy for 30 days was found to be highly ‎effective to reduce the total serum cholesterol, triglycerides, VLDL-C [p<0.05; (Table 1 and ‎Figure 1 C)]. Elevated triglyceride level is an independent risk factor for atherosclerosis ‎‎(Beatriz et al., 2011). Triglyceride rich lipoproteins (TRLs) have apo C-III that stimulates ‎activation of pro-inflammatory transcription factors nuclear factor-κB, processes ultimately ‎leads to atherosclerosis (Kawakami et al., 2006 ▶). Remnant species of TRLs can easily accumulate ‎in endothelial cells and taken up by macrophage to form foam cells similar to oxidized LDL-‎C (Gianturco et al., 1998 ▶; Botham et al., 2007 ▶). Foam cell promotes the formation of fatty streak in ‎blood vessels that is an early marker of atherosclerosis (Beatriz et al., 2011). TRLs block ‎sterol efflux from monocytes and macrophages which may diminish the protective effect of ‎HDL-C (Palmer et al., 2004 ▶; Patel et al., 2009 ▶).‎

Biochemical analysis of Cow urine proved to have many constituents; ‎copper, kallikrein, urokinase, nitrogen, uric acid, hippuric acid, phosphate and others (Jain et ‎al., 2010 ▶). Both, dietary and serum copper were inversely associated with fasting glucose, ‎total cholesterol, and LDL-C according to Bo S et al (Bo et al., 2008 ▶). Previous study also ‎indicated that the supplementation of moderate dietary copper inhibits atherogenesis in the ‎cholesterol-fed rabbit (Lamb et al., 2001 ▶), thus the copper in CUA may be responsible for its ‎lipid lowering activity.‎

Oxidative stress and free radical induced injuries play a major role in the ‎pathogenesis of a number of diseases. Hyperlipidemia produces lots of free radicals and ‎oxidative stress in blood vessels along with atherosclerosis progression, and it endangers ‎vital organs such as liver, kidney, heart and brain (Suanarunsawat et al., 2011 ▶). Augmentation of ‎serum levels of AST, ALT, AP, LDH, and CK-MB suggested suppressed cardiac and ‎hepatic functions, due to the retention of lipid in liver and heart in sham control (Table 3 and ‎Figure 1 B). CUA treatment decreased serum level of ALT, AST and LDH (p < 0.05), and improvement in hepatocytes histopathologically also seen (Table 3 and Figure 1 C). CUA has ‎many volatile fatty acids; acetic acid 2 propenyl ester, acetic acid methyl ester, 2, 2, 3 ‎trichloro propionic acid, butanoic acid-3methyl, propyl ester, 1H indol-3-acetate, acetic acid ‎phenyl ester and quinoline. They are responsible for its antioxidant action which is confirmed ‎by the estimation of thiobarbituric acid, ascorbic acid, DPPH radical scavenging activity and ‎ABTS assay (Sachdev et al., 2012 ▶). Hence, the antioxidant activity of CUA might be ‎responsible for the cytoprotective action that was found in our study. ‎

Several studies reported that residual cardiovascular risk is still apparent in ‎spite of intensive statin therapy. Residual cardiovascular risk with 80 mg atorvastatin was ‎‎22.4%, 12% and 8.7%, reported in the PROVE IT-TIMI study, the IDEAL study and the ‎TNT study, respectively (Cannon et al., 2004 ▶; LaRosa et al., 2005 ▶; Pedersen et al., 2005 ▶). ‎Residual cardiovascular risk in patients that were treated with statins is attributed to the triglycerides and ‎low HDL-C which is predominantly higher among diabetics than non diabetics (Sampson et al., ‎‎2012 ▶). Diabetes has a triad of lipid abnormality, including high levels of triglycerides, low ‎levels of HDL-C and small, dense LDL-C (Fruchart, 2013 ▶). CUA increases HDL-C as well ‎as reducing triglycerides (Table 1). These findings are in accordance with the previous studies of ‎cow urine distillate in diabetic wistar albino rats (Gururaja et al., 2011 ▶). Thus, CUA may be ‎useful as an add on therapy to reduce statin related residual CV risk in diabetic patients.‎

Metabolic syndrome is a group of interrelated metabolic abnormalities that includes ‎insulin resistance, diabetes, elevated blood pressure, obesity and dyslipidaemia (Li et al., ‎‎2013 ▶). CUA has lipid lowering, antidiabetic and antioxidant activities (Sachdev et al., ‎‎2012 ▶). Cow urine contains copper (Jain et al., 2010 ▶); while previous studies revealed an inverse ‎association between diastolic blood pressure and dietary copper intake (Bo et al., 2008 ▶). ‎Cow urine has a diuretic action which might be due to nitrogen, uric acid, phosphates and hippuric‎acid (Jain et al., 2010 ▶), Therefore cow urine may be effective as an antihypertensive that has the potential to‎ decrease all the metabolic abnormalities and also a possible ‎implication in metabolic syndrome.‎

Hence, the present study revealed that CUA has lipid lowering and ‎cytoprotective effects that may be implicated in metabolic syndrome. It is vital to mention that this study has ‎several limitations since we did not evaluate molecular mechanism, objective evidence for ‎atherosclerosis and metabolic syndrome.‎

From the current study, we concluded that CUA reduces triglycerides, ‎improves HDL-C and hepatoprotective in animals with high fat diet; therefore it may be useful in ‎diabetic dyslipidemic patients.‎
